# Embryo and Developmental Toxicity of Cefazolin Sodium Impurities in Zebrafish

**DOI:** 10.3389/fphar.2017.00403

**Published:** 2017-06-26

**Authors:** Bo Chen, Zhu-Qing Gao, Ying Liu, Yang-Min Zheng, Ying Han, Jing-Pu Zhang, Chang-Qin Hu

**Affiliations:** ^1^Department of Pharmacology, Institute of Medicinal Biotechnology, Chinese Academy of Medical Sciences and Peking Union Medical CollegeBeijing, China; ^2^Division of Antibiotics, National Institutes for Food and Drug Control, Graduate School of Peking Union Medical CollegeBeijing, China

**Keywords:** cefazolin, impurities, structure-toxicity correlation, toxicity evaluation, zebrafish

## Abstract

Cefazolin sodium is an essential drug that is widely used in clinical therapy for certain infective diseases caused by bacteria. As drug impurities are considered to be one of the most important causes of drug safety issues, we studied embryotoxicity, cardiotoxicity, and neurotoxicity of nine cefazolin sodium impurities in zebrafish embryo and larvae for the objective control of impurity profiling. LC-MS/MS was employed to analyze the compound absorbance *in vivo*, and the structure-toxicity relationship was approached. Our results suggested that the structure of MMTD (2-mercapto-5-methyl-1, 3, 4-thiadiazole) is the main toxic functional group for embryo deformities; the 7-ACA (7-aminocephalosporanic acid) structure mainly affects motor nerve function; and both the MMTD and 7-ACA structures are responsible for cardiac effects. Impurity G (7-ACA) presented with the strongest toxicity; impurity A was most extensively absorbed to embryo and larvae; and impurity F (MMTD) exhibited the strongest apparent toxic effect; Therefore, impurities F and G should be monitored from the cefazolin sodium preparations.

## Introduction

Cephalosporin has the characteristics of a wide-spectrum antibacterial drug, low toxicity, and resistance to penicillin enzymes. In addition, cephalosporin accounts for about 50% of the total sales of antibiotics in China; of this, 2% of the sales involve cefazolin sodium (unpublished data). A wide range of clinical applications has led to the continuous emergence of adverse drug reactions (ADRs) caused by cephalosporin. Fan et al. analyzed 1,169 cases of cephalosporin-induced ADRs that were reported from 1998 to 2010 in 958 publications in China by using the bibliometrics method: the most frequent were systemic reactions (35.67%) followed by central and peripheral nervous system reactions (19.76%), and the occurrence of blood abnormality (13.94%). The main clinical manifestations of ADRs were anaphylactic shock (28.14%), disulfiram-like reactions (12.74%), and hematuria (8.04%). Of the 1,169 cases, 118 (10.09%) were caused by cefazolin sodium (Fan, 2012). The familiar ADRs of cefazolin sodium include not only the common ADRs of cephalosporins (He, 2004; CFDA, 2014), but relatively high proportions of cardiovascular system reactions (16/152, 7.2%) and nervous system reactions (8/152, 5.3%) (He, 2004). Arrhythmia is the most common cardiovascular adverse reaction caused by beta-lactam antibiotics (73.85%) (Zhang et al., 2013b). The nervous system adverse reactions are considered to be from cephalosporin, which inhibits the combination of gamma aminobutyric acid to its receptors, blocks the synthesis and transport of neurotransmitter amino acids, suppresses the $$Na + -K + -ATP enzyme activity of central nervous cells, and reduces the resting membrane potential, leading to CNS toxicity reactions (Grill and Maganti, 2008).

**Figure 1:**
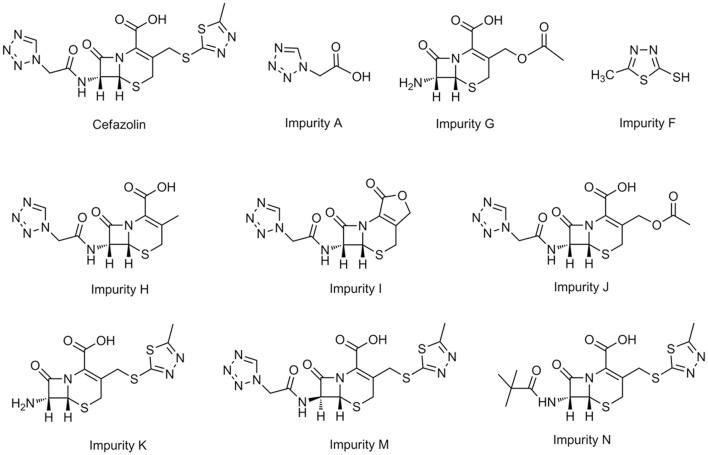
Structures of Cefazolin and related impurities.

Drug impurities are considered to be one of the important causes of drug safety issues (Alsante et al., 2014). The types and content of organic impurities in drugs are generally referred to as impurity profile. Currently, the impurity profile is still a research highlight of drug quality control (Jain and Basniwal, 2013; Narayanam et al., 2013; Maggio et al., 2014). According to the International Conference on Harmonization (ICH), if the impurity content is larger than the qualification threshold (for chemical drugs the threshold is 0.15%) in the research and development of the new drug, a toxicity assessment should be performed (ICH, 2006). An ideal concept of a drug quality control profile should be aimed at each of the impurities in a drug, which means that the quality control limit of each impurity should be set up based upon its corresponding physiological activity (Görög, 2006). Recent years, zebrafish has been used as valid models to evaluate drug toxicity (Barros et al., 2008; Planchart et al., 2016), including embryotoxicity models (Zhang et al., 2010, 2013a; Lantz-McPeak et al., 2015), cardiac toxicity models(Milan et al., 2006; Macrae, 2010; Han et al., 2015; Sarmah and Marrs, 2016), neurotoxicity models (Tierney, 2011; Selderslaghs et al., 2013). We assessed the harmfulness of the drug impurities by evaluating the relative toxicity between drug impurity and active pharmaceutical ingredients (APIs; Wang et al., 2010; Zhang et al., 2012; Sun et al., 2014); LC-MS/MS was employed for analyzing drug content in zebrafish bodies to exclude the drug absorption effect upon the organism response (Zhang et al., 2014). Based upon the above models and methods, nine types of known impurities of cefazolin were divided into three groups: the first group included impurity A [tetrazol eacetic acid (TAA), the 7-side chain of cefazolin sodium], impurity G (7-ACA, the precursor of cefazolin), and impurity F (MMTD, the 3-side chain of cefazolin sodium), which are the basic structural units of cefazolin sodium. The second group included impurity H (Cefazolin 3-methyl analog), impurity I (Cefazolin lactone), and impurity J (Cefazolin acetoxy analog); the 7-side chain of them is the same as cefazolin sodium, but the 3-side chain is different. The third group included impurity K (Cefazolin deacylated), impurity M (Cefazolin epimer), and impurity N (Cefazolin pivaloyl); their 3-side chain is the same as cefazolin sodium, but their structure or the configuration of their 7-side chain is different (Figure 1). We evaluated the embryo toxicity, the neurobehavioral toxicity, and the cardiac toxicity of the impurities, to hopefully provide some data for an objective evaluation of the impurity profiling control of cefazolin sodium in the current pharmacopeias of various countries.

## Materials and methods

### Samples

Cefazolin reference substance and nine kinds of the impurity reference substances were provided by the National Institutes for Food and Drug Control of China. The structure of each impurity was confirmed by MS and NMR; the HPLC normalized purity was >93%.

### Toxicity study in zebrafish

Zebrafish (*Danio rerio*, Tuebingen) were fed at the Institute of Medicinal Biotechnology, Chinese Academy of Medical Sciences, Beijing Union Medical College. All experimental protocols were approved by and performed in accordance with the Good Laboratory Practice regulations for non-clinical laboratory studies of drugs issued by the National Scientific and Technologic Committee of the People's Republic of China.

### Zebrafish embryo/larvae treatment by cefazolin and nine kinds of the impurity reference substances

According to Zhang et al. (2014), zebrafish breeding water was prepared with Instant Ocean sea salt (CNSG Tianjin, China). Zebrafish wild type (WT) embryos at 6 hpf and larvae at 3 dpf were used with 30 embryos or 30 larvae each petri dish. Both embryos and larvae were immersed, respectively, in 3–4 mL of the drug testing solutions (drugs were dissolved in breeding water) in a 20-mm dish for 3 days (organogenesis at period of 6–72 hpf), but the solutions for the treatment of the larvae were replaced every 24 h, and zebrafish were observed under a microscope. Three or four concentrations for the administered groups were used for each compound. Abnormal phenotypes and mortality from each treatment group were documented for 3 sequential observational days. Zebrafish WT embryos (*n* = 30) that were incubated in the breeding water were the controls and each experiment was repeated at least three times. Anesthetic MS-222 (3-aminobenzoic acid ethylester, methanesulfonate salt, Sigma) was added to the larval solution to 0.016% final concentration and then the zebrafish bodies (30 each dish) were washed three times with deionized water, triturated in 100 μL of deionized water and followed a centrifugal separation; then the supernates were used for the measurement of the compounds *in vivo*. Values for the 10% teratogenic concentration (TD_10_), 50% teratogenic concentration (TD_50_) and 50% lethal concentration (LD_50_) were calculated using a Bliss algorithm.

### Zebrafish neurons toxicity test

According to Hu and Zhang (2014), 5 dpf zebrafish larvae (*n* = 30) were exposed to testing solution for 1 day, then the larvae with no obvious malformations were used for further analysis. The larvae were placed, respectively, in each micropore of a 48-well plate, with 8 in each group, for behavior to be detected by instrument of the ZEBRALAB (Viewpiont, ZebraLab 3. 3. France), setting it to record every 10 s under specific conditions: (1) in the dark it continuously recorded for 20 min; and (2) under a bright/dark cycle (darkness for 5 min and light stimulation for 10 s) it recorded for 4 cycles. Untreated larvae were used as a normal control. The larvae swimming motions were observed and the distance and speed of movement were recorded and analyzed. Speeds lower than 0.2 cm/s were defined as inactive motion; speeds faster than 0.2 and lower than 4.0 cm/s were defined as medium-speed motion, and speeds >4.0 cm/s were defined as high-speed motion.

### Zebrafish heart rate test

According to Tong et al. (2011), 2 dpf zebrafish embryos, with 20 in each group, were exposed to testing solution for 1 day then the embryos with no obvious malformations were used for further testing. The heart rates (including atrial and ventricular beat frequency) from 10 larvae each group were recorded by Monochrome Digital Camera (DVC-340M) under a microscope (Olympus), with a continuous recording time for each larva of no <10 s. Using untreated 3 dpf larvae as a control, the changes in heart rate and rhythm caused by the drugs were analyzed.

### Determination of drug absorption in zebrafish by LC-MS/MS

Drug contents in the embryos that were exposed to drugs from 6 hpf to 3 dpf were regarded as the absorption characterization of a drug in the zebrafish embryo toxicity test and in the cardiac toxicity test. The drug amount in the larvae exposed to drugs from 3 dpf to 5 dpf represented the absorption characterization of a drug in zebrafish neural toxicity. We then detected levels of the compounds *in vivo* in the 2 drug-administering durations.

### LC-MS/MS method

The liquid chromatography system was a Prominence UFLCXR LC with autosampler (Shimadzu, Tokyo, Japan), equipped with a CAPCELL PAK C18 MG S5 column (2.0 mm i.d. × 150 mm) (Shiseido Scientific Instruments, Tokyo, Japan). The mass spectrometer was an AB 6500 Q-Trap (ABSciex, Framingham, MA) equipped with electrospray ionization (ESI), operated in the positive ion or negative ion mode, and quantification was performed by multiple reaction monitoring (MRM) scanning modes. Mobile phase A contained 5 mmol/L of ammonium acetate solution and mobile phase B was acetonitrile. The temperature of the autosampler was set at 4°C and the column temperature was 30°C. Chromatographic conditions are shown in Table 1; condition 1 was used for the positive mode and condition 2 was used for the negative mode. MS conditions are listed in Table 2; the compound parameters, including the declustering potential (DP), collision energy (CE), cell exit potential (CXP), and the ion source parameters were as follows: curtain gas (CUR), ion spray voltage (IS), temperature, nebulizer gas (GS1), and turbo gas (GS2).

**Table 1:** HPLC conditions for LC-MS/MS.

**Compound**	**Time (min)**	**Flow velocity (μL/min)**	**Mobile phase B (%)**	**Mobile phase A (%)**
Cefazolin	0	600	1	99
impurity G	6.0	600	30	70
impurity H	8.0	600	80	20
impurity I	10.0	600	80	20
impurity J	10.1	600	1	99
impurity K	15.0	600	1	99
impurity M				
impurity N				
impurity A	0	600	5	95
impurity F	10.0	600	5	95

**Table 2:** MS conditions for compound detection.

**Compound**	**Ion mode**	**Parameters**									
		**Compound**					**Ion source**				
		**m/z**	**MRM paris**	**DP**	**CE**	**CXP**	**CUR**	**IS**	**TEM**	**GS1**	**GS2**
Cefazolin	positive	455	→ 323	31	21	11	40	5,500	550	40	40
			→ 295		23	8					
			→ 156		16	9					
Impurity G	positive	295	→ 235	30	11	2	40	5,500	550	40	40
			→ 207		15	2					
Impurity H	positive	325	→ 282	50	14	3	40	5,500	550	40	40
			→ 158		12	2					
Impurity I	positive	323	→ 280	70	14	2	40	5,500	550	40	40
			→ 156		18	4					
			→ 112		120	3					
Impurity J	positive	400	→ 323	15	15	2	40	5,500	550	40	40
			→ 295		22	3					
								→ 156		23	2
Impurity K	positive	345	→ 185	31	15	5	40	5,500	550	40	40
			→ 167		24	5					
			→ 141		29	7					
Impurity M	positive	455	→ 323	31	21	11	40	5,500	550	40	40
			→ 295		23	8					
			→ 156		16	9					
Impurity N	positive	429	→ 297	40	14	6	40	5,500	550	40	40
			→ 269		24	5					
			→ 142		25	7					
Clenbuterol (internal standard)	positive	277	→ 203	43	23	11	40	5,500	550	40	40
			→ 168		40	8					
Impurity A	negative	127	→ 83	−45	−14	-7	40	−4,500	550	40	40
			→ 59		−20	−5					
Impurity F	negative	131	→ 101	−20	−14	−7	40	−4,500	550	40	40
			→ 58		−23	−4					
Benzoic acidin (internal standard)	negative	121	→ 77	−18	−16	−4	40	−4,500	550	40	40

### Sample preparation

Before zebrafish collected, MS-222 (0.016% final concentration) was used for larval anesthesia, and then the zebrafish were washed three times with deionized water, collected in a tube (30 larvae each tube) and triturated with 100 μL of deionized water for 2 min. According to Zhang et al. (2014), 10 μL of the internal standard (10 ng/mL clenbuterol in the positive ion mode or 20 μg/ml benzoic acid in the negative ion mode) was added to 100 μL of zebrafish homogenate samples. Protein was removed from the samples by vortexing them with 300 μL of methanol for ~2 min. The samples were centrifuged for 10 min at 10,000 rpm and 10 μL of the supernatant was injected into the LC-MS/MS system.

### Method validation

Seven-level standard solutions spiked into blank zebrafish homogenate were used to construct the calibration curves. The linear ranges of each compound are listed in Table 3. Method validation was completed according to guidelines for the validation of bioanalytical methods (US Department of Health and Human Services, 2001; CHMP, 2012). The intra- and inter-day precision values did not exceed ±15% and the stability was within ±15%.

**Table 3:** Standard curves and ranges of Cefazolin and its related impurities.

**Compounds**	**Standard curve**	**Range (μg/ml)**	**Correlation coefficient (r)**
Cefazolin	y = 13.9x + 0.0525	0.05–10	0.9998
Impurity A	y = 0.49x + 0.0117	0.2–10	0.9959
Impurity F	y = 0.149x + 0.00868	0.2–10	0.9997
Impurity G	y = 2.75x + 0.081	0.05–10	0.9971
Impurity H	y = 1.37x + 0.00039	0.05–10	0.9995
Impurity J	y = 4.09x + 0.00847	0.05–10	0.9995
Impurity K	y = 20.9x + 0.0499	0.05–10	0.9938
Impurity M	y = 4.69x + 0.00187	0.05–10	0.9994
Impurity N	y = 57x + 0.38	0.05–10	0.9992

## Results

### Comparison of toxicity of cefazolin impurities in zebrafish embryonic development

**Figure 2:**
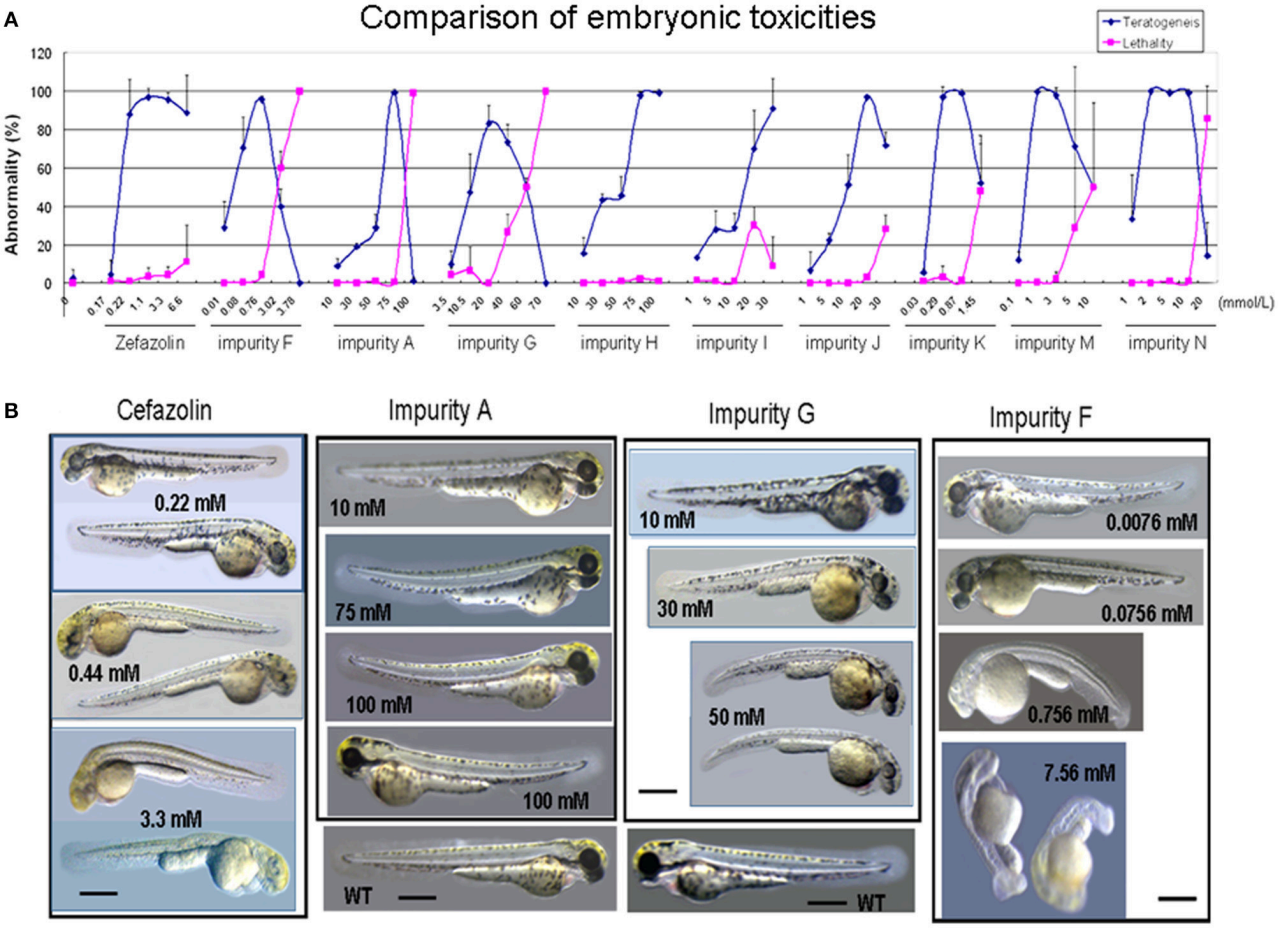
Effect of Cefazolin and its impurities on zebrafish embryotoxicity. **(A)** Comparison of effects of Cefazolin and its impurities A, F and G on zebrafish embryonic development in teratogenesis and lethality. **(B)** Abnormal Phenotypes of zebrafish embryos treated by Cefazolin and its impurities A, F, and G. WT, a wild type embryo as a normal control. The scale bars indicate 430 μm.

The teratogenic rate, mortality, and abnormal phenotypes of the zebrafish caused by cefazolin and its impurities during embryo development are shown in Figure 2. Further analysis of the correlation between the structures and toxicity can be seen in Table 4. The first group of impurities had the basic structural unit of cefazolin sodium. The abnormal phenotypes of impurity F (the 3-side chain structure unit MMTD) are as follows: the body becomes transparent and yellow, the body length shortens, the axis bends, and the embryonic notochord is seriously twisted in an “S” shape. The teratogenic phenotypes of impurity A (the 7-side chain structure unit TAA) are pericardial edema, blood pool, and slight trunk bending. The teratogenic phenotypes of impurity G (the mother ring structure, 7-ACA) are the smaller head and eyes, poor transparency, and the short body.

**Table 4:** Comparison of cefazolin impurities in embryotoxicity testing of zebrafish.

**Compound**		**Administration concentration**				**Concentration *in vivo*^†^ (RSD) (mmol/L)**	**Body burden (mmol/larva^†^)**	**Teratogenic phenotypes**
		**TD_10_ (mmol/L)**	**TD_50_ (mmol/L)**	**LD_10_ (mmol/L)**	**LD_50_ (mmol/L)**			
Cefazolin		0.06	0.19	7.2	>100^*^	2.1 × 10^−5^ (17.5)	6.8 × 10^−11^	Yellow and transparent body, shortened body length, bent anterior-posterior axis, twisted notochord, pericardial sac expands, bloodless and rope-like heart, and opaque belly.
The first group of impurities	Impurity F	0.002	0.03	1.1	2.3	4.5 × 10^−4^ (6.3)	1.5 × 10^−9^	Yellow and transparent body, shortened body length, bent anterior-posterior axis, seriously twisted notochord in an “S” shape, pericardial sac expands, bloodless heart with a slow heart rate, no melanin spots, colorless eyes, opaque yolk sac and extension structure.
	Impurity G (7-ACA)	2.6	9.3	16.8	48.7	1.5 × 10^−4^ (2.9)	5.0 × 10^−10^	Smaller head and eyes, poor transparency, and shortened body.
	Impurity A (TAA)	16.2	34.6	~60^*^	75 < LD_50_ <100^*^	2.4 × 10^−2^ (2.8)	8.1 × 10^−8^	Mild pericardial edema, mild congestion causing blood pooling, and slightly bent trunk.
The second group of impurities	Impurity H	10.4	20.6	/	/	4.6 × 10^−4^ (5.3)	1.5 × 10^−9^	Slightly smaller head, eyes, shorter body, pericardial edema, mild congestion causing blood pooling.
	Impurity I	1.5	8.9	17.1	/	drug decomposition	/	Slightly shrunken head and eyes, pericardial edema, mild congestion causing blood pooling.
	Impurity J	1.8	6.1	~20^*^	~45^*^	1.3 × 10^−4^ (9.7)	4.3 × 10^−10^	Under a low concentration, head and eyes slightly shrunken, body length slightly shortened, pericardial edema, mild congestion causing blood pooling; under a high concentration, abnormal phenomena aggravated, stubby yolk sac extension structure, and bent trunk in a few of the larvae.
The third group of impurities	Impurity K	~0.1	0.03 < TD_50_ <0.3^*^	0.75	~1.5^*^	8.1 × 10^−4^ (8.2)	2.7 × 10^−9^	Yellow body surface, “S” shaped yellow body and notochord, bent tail; some embryos showed congestion causing blood pooling, eye edging, ventral tail, shortened body, and opaque abdomen.
	Impurity M	~0.03	0.1 < TD_50_ <1^*^	3.5	8.5	4.9 × 10^−5^ (10.9)	1.6 × 10^−10^	Slightly shortened body length, bent body axis, poor body transparency, less melanin, pericardial edema, and congested blood pooling.
	Impurity N	~0.75	1 ≤ TD_50_ <2^*^	~10^*^	10 < LD_50_ <20^*^	5.4 × 10^−4^ (8.2)	1.8 × 10^−9^	Yellow body, less or no melanin, pericardial edema, congestion and invagination causing blood pooling, cardiac abnormalities, smaller abdomen, and thicker yolk extension.

*/, LD50 was not observed, or impurity in vivo was not determined*.

* *Estimation from measured values*;

† *50% of the embryos had toxic reactions (teratogenic + lethal); ^‡^ mmols/larva = concentration(mmol/L) × 100 μL/30 (zebrafish number) × 10^−6^*.

Drug concentration *in vivo* was analyzed as 50% of the embryos showed a toxic reaction. The toxic concentration of 50% (TD_50_) of impurity A was 1,000 times higher than impurity F. The *in vivo* drug concentration of impurity A was about 50 times higher than impurity F under TD_50_ concentration exposures, indicating that impurity F was more easily absorbed by the embryo than impurity A and its toxicity was about 50 times higher than impurity A. Similarly, the TD_50_ of impurity G was about 300 times greater than impurity F, but it's *in vivo* drug concentration was similar to impurity F, suggesting that “low toxicity” of impurity G in the embryo testing probably resulted from being less easily absorbed than impurity F. Based on Table 4, under TD_50_ concentration exposures, the order of contents *in vivo* is impurity A > impurity F > impurity G, and the order of absorption capacity *in vivo* is impurity F > impurity A > impurity G in the group 1 impurities. The results indicated that the impurities F is more potent embryotoxins than other impurities and impurities G is clearly less potent than other impurities.

The toxic reaction of the second groups of impurities was mainly teratogenic and their characteristic appearance was more or less like that of impurity G (head and eyes slightly shrunken, and body length slightly shortened) and like that of the impurity A (pericardial edema, mild congestion, and blood pooling) suggesting the structures of 7-ACA and TAA as two different toxic functional groups work together in producing toxic reactions in this impurity group.

The TD_50_ of impurity H was about 3.5 times of impurity J, also its *in vivo* content was about 3.5 times as much as impurity J, suggesting that the absorption capacity of both were equivalent. The toxicity of impurity J was about 3.5 times as much as impurity H, which means the acetoxyl group at the 3-side chain of 7-ACA substituted by the methyl group slightly reduces the toxicity. The TD_50_ and drug *in vivo* content of impurity J and impurity G were both equivalent, so the 7-site of the 7-ACA connecting to TAA had no effect on the absorption. Because impurity I (cefazolin lactone) was easily hydrolyzed in an aqueous solution, it was not detected *in vivo*.

All zebrafish treated by the third group of impurities showed characteristics of impurity F at different levels (impurity F had body length shortening and anterior-posterior axis bending) though they showed other differential abnormal phenotypes (Table 4). The abnormal phenotype and *in vivo* content of impurity K were very similar to impurity F, suggesting that the MMTD structure was the main teratogenic functional group for impurity K. The teratogenic effect of cefazolin (7R configuration) presented the typical impurity F phenotype (transparent yellow body, shortened body length, bent anterior-posterior axis, seriously distorted notochord in an “S” shape (Zhang et al., 2010). However, cefazolin 7S-isomers (impurity M) not only showed typical teratogenic phenotypes of MMTD, but it also presented with part of the teratogenic phenotype of TAA (mild pericardial edema and congestion of blood pooling), implying that the configuration of the 7-side chain of cephalosporin can affect the toxicity of the toxic functional groups. Based on the body burden in Table 4, orders of their *in vivo* absorption capacity are as follows: impurity K > impurity N > impurity M. The results also indicated that the potent embryotoxin of impurity K is similar to impurity F.

### Comparison of toxicity of cefazolin impurities in zebrafish larvae testing

No malformation was observed at every concentration of cefazolin and its impurities in the larval administration tests because the main organs of zebrafish are developed before 72 hpf (Grunwald and Eisen, 2002). However, larvae death was observed at 5 dpf with the increase of concentrations. The LD_50_ and *in vivo* content of impurities in zebrafish larvae are shown in Table 5. Compared with the TD_50_ in Table 4, the LD_50_ of the majority of the compounds in the 3 dpf groups was significantly lower than that of the 6 hpf groups; however, the *in vivo* drug concentration of the two groups were almost at the same level.

**Table 5:** Comparison of toxicities of Cefazolin impurities in zebrafish larvae testing.

**Compounds**		**LD_10_ (mmol/L)**	**LD_50_ (mmol/L)**	**Test concentrations (mmol/L)**	Concentrations in vivo^*^ (mmol/L) (RSD)	**Body burden (mmol/larva^‡^)**
Cefazolin		0.18	0.26	0.11	1.5 × 10^−5^ (10.0)	5.1 × 10^−11^
	0.26			2.1 × 10^−5^ (12.1)	6.8 × 10^−11^	
First group of impurities	Impurity A	1.95	3.85	0.04	7.3 × 10^−4^ (10.0)	2.4 × 10^−9^
				3.85	0.14 (8.4)	4.7 × 10^−7^
	Impurity F	0.07	0.11	0.04	7.6 × 10^−3^ (3.5)	2.5 × 10^−8^
				0.11	3.3 × 10^−2^ (1.4)	1.1 × 10^−7^
	Impurity G	0.88	1.32	0.18	2.2 × 10^−5^ (4.0)	7.2 × 10^−11^
				0.74	3.1 × 10^−5^ (2.7)	1.0 × 10^−10^
Second group of impurities	Impurity H	0.04	0.22	0.11	8.7 × 10^−5^ (12.0)	2.9 × 10^−10^
				0.22	1.4 × 10^−4^ (20.0)	4.5 × 10^−10^
	Impurity I	0.06	0.08	0.08	/	1.5 × 10^−10^
	Impurity J	~0.05	/	0.31	4.5 × 10^−5^ (8.2)	2.0 × 10^−10^
				0.52	6.0 × 10^−5^ (7.5)	4.8 × 10^−10^
Third group of impurities	Impurity K	0.19	0.37	0.15	1.4 × 10^−4^ (5.3)	1.2 × 10^−8^
				0.37	3.6 × 10^−3^ (6.1)	2.9 × 10^−10^
	Impurity M	0.06	0.12	0.07	2.5 × 10^−4^ (7.5)	8.4 × 10^−10^
				0.12	6.4 × 10^−4^ (5.6)	2.1 × 10^−9^
	Impurity N	0.03	0.12	0.07	9.2 × 10^−4^ (8.3)	3.1 × 10^−9^
				0.12	2.1 × 10^−3^ (11.6)	6.7 × 10^−9^

*/, LD_50_ was not observed, or impurity in vivo was not determined*.

* *50% of the embryos had toxic reactions (teratogenic + lethal)*;

‡ *mmol/larva = concentration(mmol/L) × 100 μL/30 (zebrafish number) × 10^−6^*.

### Effects of cefazolin sodium impurities on larval zebrafish motor nerve system

**Figure 3:**
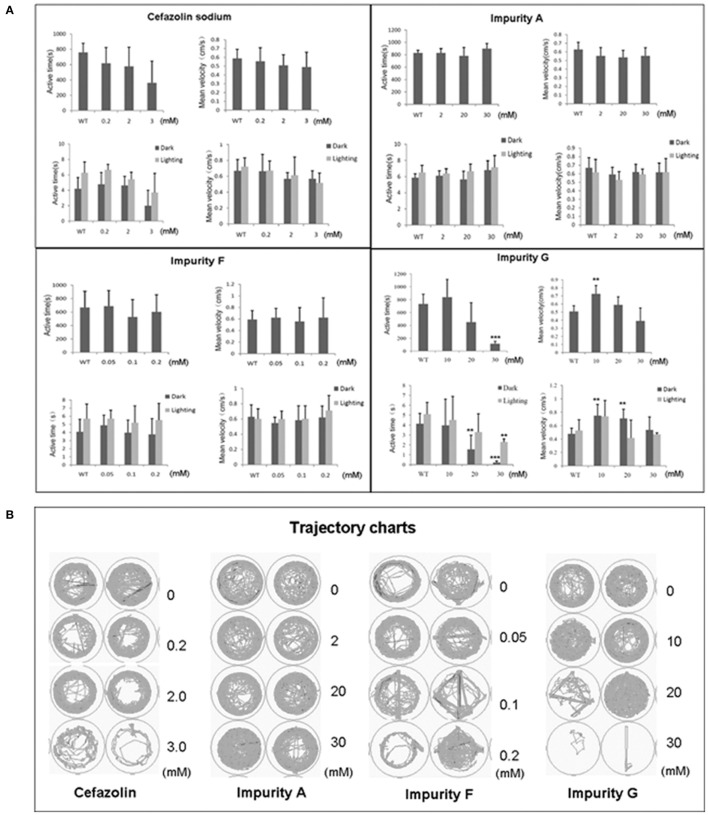
Impacts of cefazolin and its impurities on the motor nerve system of zebrafish larvae. **(A)** Show the statistical results of the locomotion behavior of zebrafish larvae in their swim time and swim speeds when they were exposed to cefazolin or its three impurities A, F, and G, respectively. **(B)** Typecal trajectory charts of zebrafish larvae treated by cefazolin or its three impurities A, F, and G at a series of concentrations.

Zebrafish larvae at 5 dpf were exposed to the testing solution for 1 day then the larvae with no obvious malformations were used to test zebrafish locomotion behavior. Based on the influence of cefazolin and its impurities on the motor nerve system (Figure 3) combined with the absorption levels of different compounds in zebrafish (Table 5), we analyzed the neurotoxicity of cefazolin and its impurities.

For the first group of impurities, under dark conditions or under light/dark periodic stimulation, the time of larval active movement was shortened in impurity G in a concentration-dependent manner, and the larvae swimming velocity regularly increased at first and then decreased; while impurities A and F did not experience the same phenomena (Figures 3A,B). Analysis of the compound concentration *in vivo* showed that impurities A and F were more easily absorbed into zebrafish larvae than impurity G; in other words, the impact of impurity G on larvae active movement time and speed did not result from its higher *in vivo* concentration than impurities A and F. This evidence suggests that the 7-ACA (impurity G) structure may be a major toxic function group that impacts the motor nerve function of zebrafish.

In the second group of impurities, impurity I had no obvious effect on active movement time and the velocity of larvae under both conditions of dark environment and the periodic dark/light switch stimulation (data not shown). Because impurity I can be rapidly decomposed in an aqueous solution, its concentration in the body was not detected. Impurity J, under both dark conditions and periodic dark/light stimulus, resulted in a decreased time in larval active movements in a concentration-dependent manner, but its influence on speed was not obvious. Impurity H had just slightly increased active movement time of larvae in the absence of light stimulation conditions, its effect on speed was not obvious; while the active time and speed of larval movement in the periodic occulting stimulus had not been observed. Under the similarly treated concentrations, the *in vivo* concentration of impurity H was about 10 times higher than that of impurity J, suggesting that the acetoxy group at the 3-side chain plays an important role in the active movement of zebrafish larvae. Further, compared with the results of impurity G (7-ACA), it can be implied that the combination of TAA with the 7-side chains of 7-ACA can decrease the impact on the locomotive nervous system of zebrafish larvae.

With the third groups of impurities, in both the absence of light stimulation and periodic occulting stimulus conditions, impurities M and N could cause active time and speed of larval movement slightly increase at first and then decreased to the level of the normal control group (data not shown). However, cefazolin sodium significantly decreased the active movement time of larvae in a concentration-dependent manner, but the impact on the speed of movement was moderate (Figure 3A). Impurity K has no 7-side chain, and the structure difference between impurities M and N is their 7-side chains, but impurity K has no significant difference from impurities M and N *in vivo* concentrations, and their impact on the motor nerve system of zebrafish larvae are similar, too, which suggests the 7-side chain structure of cefazolin is not an important functional group for the nerve motor system of zebrafish larvae. In addition, impurity M and cefazolin are R/S isomers of each other, and the difference of the two compounds in concentration *in vivo* is about 10 times, suggesting that the spatial configuration of the 7-side chain of cephalosporin can influence the toxic effects of 7-ACA functional group by affecting *in vivo* absorption of the compounds. In summary, the neurotoxic effects of functional groups of cefazolins are mainly correlated to their mother nucleus structure of cephalosporin. The effects of impurities J and H (the acetoxy group at the 3-side chain of 7-ACA is substituted by the methyl group) on the motor nerve system of zebrafish larvae are different and the impacts of the two are also different from impurities M and N (containing the MMTD structure at the 3-side chain), suggesting that the 7-ACA mother nucleus and its 3-side chain jointly play a role on the motor nerve system of zebrafish larvae.

### Effects of cefazolin sodium impurities on the cardiac function of zebrafish larvae

**Figure 4:**
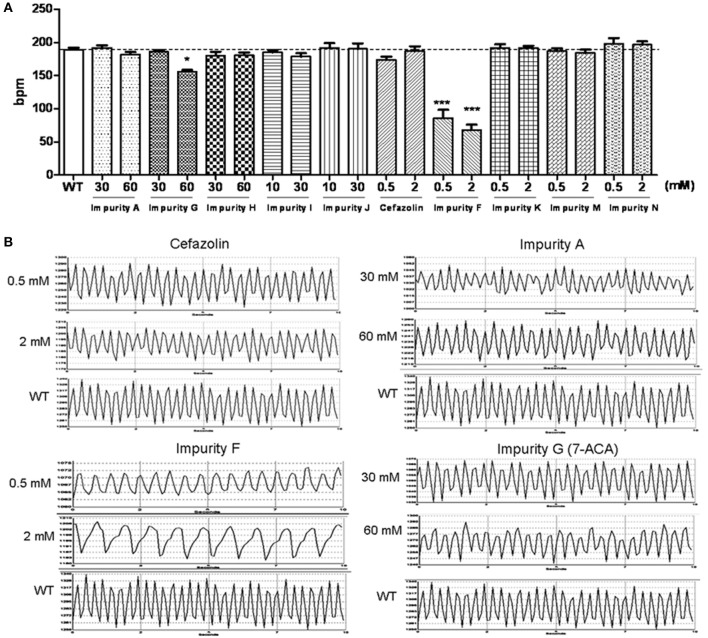
Impact of cefazolin and its impurities on heart rates of zebrafish larvae. **(A)** Comparison of effects of cefazolin and its impurity on the heart rate of zebrafish larvae. A horizontal dashed line in the histogram represents a normal heart rate of wild type larvae. **(B)** Heart rate recording charts of zebrafish larvae (3 dpf) that were administered by cefazolin and impurities F, A, and G. WT, a wild type larva as a normal control.

Zebrafish embryos at 2 dpf were exposed to the testing solution for 1 day then the larvae with no obvious malformations were employed for heart rate recording. Comparing the influence of cefazolin and its impurities on the heart rates of the embryos (Figure 4) and the absorption levels of different compounds in zebrafish larvae (Table 5), we analyzed the cardiotoxicity of cefazolin and its impurities.

In the first group of impurities, A and G slowed the heart rate of larvae slightly only at a higher concentration (60 mmol/L); while impurity F at 0.5 mmol/L led to a slowed heart rate of up to 50% or less of the normal control (Figure 4A). The *in vivo* concentration analysis of the impurities showed that impurities F and A were more easily absorbed than impurity G by the body (Table 5). Because impurity F was more easily absorbed than all of the other impurities, it was easy to observe cardiac toxicity (the heart rate recording showed that it led to atrioventricular conduction block) (Figure 4B).

Impurity H, I, and J showed no effect on embryonic heart rate at 30 mmol/L. At 60 mmol/L, for impurity H, the heart rate was reduced slightly, similar to 7-ACA, and the amplitude of the heartbeat was mildly lowered on the heart rate diagram (Figure 4B); but impurity I and J had no effect on embryonic heart rate (Figure 4A).

In comparing the impurities K, M, and N with cefazolin, there were remarkable differences in the concentration *in vivo* (impurity N was about 10 times as much as impurity M and impurity M was about 10 times as much as cefazolin, and impurity K was between the impurity N and impurity M) (Table 5). The groups of the three impurities had the similar heart rates to the control group (WT) (Figure 4A). The heart rate was slowed down at 0.5 mmol/L of cefazolin, but went back to the normal rate at 2 mmol/L. These results suggest that the contents *in vivo* of these compounds are not the key factors affecting the function of the heart.

## Discussion

The quality of a drug product is closely related with its impurities. Nowadays, there is a growing attention on the toxic events associated with drug impurities. Herein, we used zebrafish as a toxicological evaluating platform to study the toxicity of cefazolin sodium impurities. Organism absorbability of drugs or impurities and their toxic reactions vary in different development stages. Also, different developmental periods in zebrafish appeared to be differentially sensitive to the same compound. By observing the abnormal phenotype of the embryonic development and assessing the LD_50_ values, zebrafish embryos at 6 hpf are considered to be suitable for evaluating the embryonic toxicity of chemical drugs (Zhang et al., 2014). The larval malformation was not observed in the larval tests with cefazolin and its impurities, which probably were resulted from the drug exposure later than development of the main organs of zebrafish (Grunwald and Eisen, 2002). However, the larval death was observed at 5 dpf with the increase of concentrations. So, the larvae at 3 dpf are more suitable to evaluate acute toxicity of a drug.

Both absorption capacity *in vivo* and content *in vivo* of a drug or an impurity are two important factors for objective evaluation of its effect or toxicity, instead of depending on its administration concentration only. Toxicity comparison among the cefazolin impurities was performed by their contents *in vivo* with TD_50_-concentration exposure (Tables 4, 5). The results suggest that the *in vivo* drug contents at LD_50_ or TD_50_ reflect the toxic effect of the compound structure itself, and the LD_50_ or TD_50_ values reflect the integrative toxic effects of the compound toxicity and its absorption capacity. For example, in the first group of cefazolin impurities, under TD_50_ exposure, the order of contents *in vivo* is impurity A > impurity F > impurity G, but differentially, the order of absorption capacity *in vivo* is impurity F > impurity A > impurity G, which means that a compound *in vivo* absorption capacity can affect its TD_50_ value and *in vivo* contents. The above comprehensive analysis shows that impurity G had the strongest toxic effect, impurity A was most easily absorbed by the organism, and impurity F had the strongest apparent toxic effect among the cefazolin impurities.

## Conclusion

**Figure 5:**
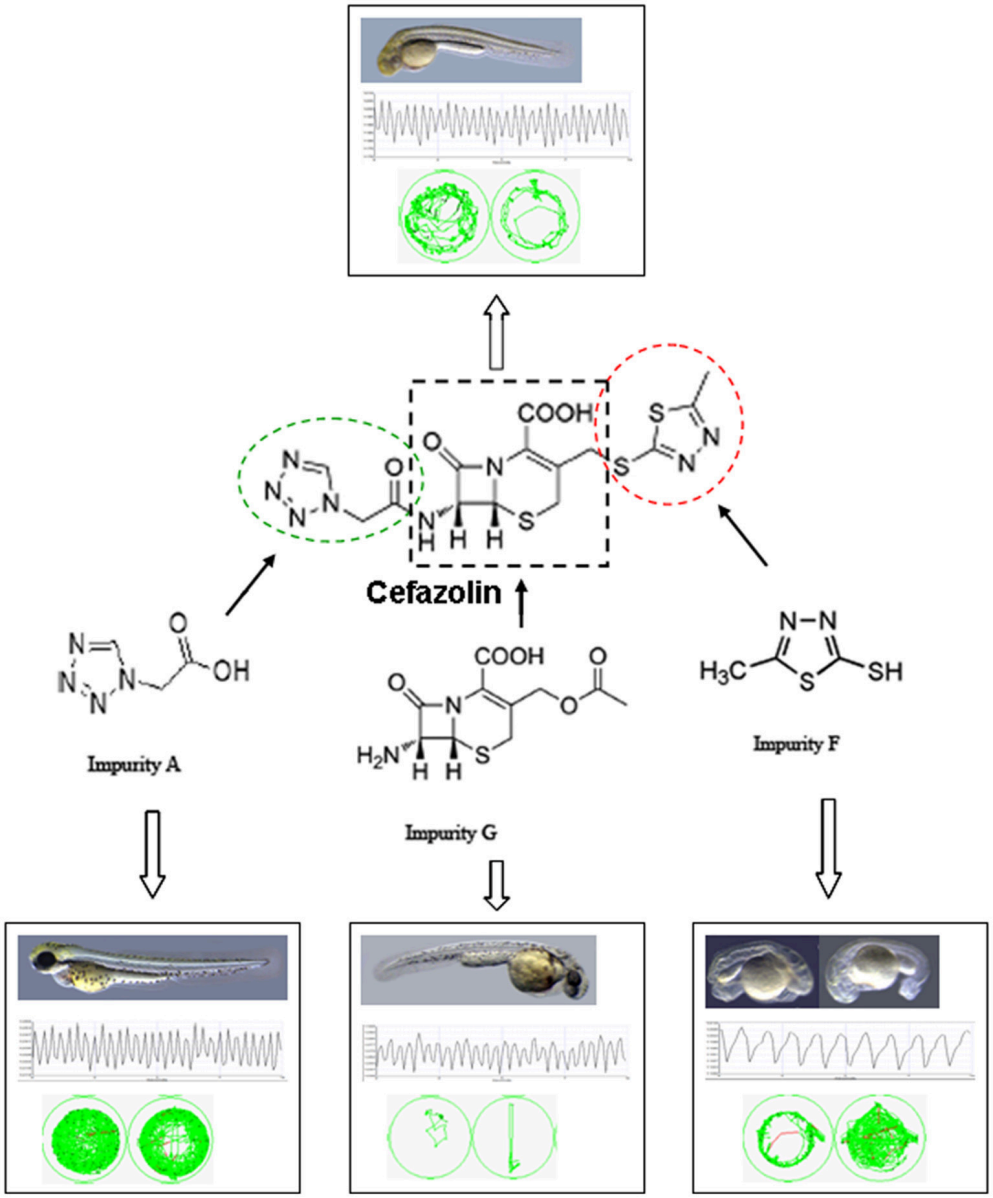
Schematic representation of the toxicity of cefazolin impurities involving in embryonic development, cardiac function, and swim behavior in zebrafish.

Using the animal model of zebrafish, we evaluated the toxicity of nine impurities of cefazolin sodium on embryonic development, neurobehavior, and cardiac function; and investigated the correlation between the structures of the impurities and toxicity reactions combined with the *in vivo* contents of the compounds. Among the impurities of cefazolin sodium, impurity G was the strongest one in toxicity, impurity A was the most easily absorbed by the body, and impurity F exhibited the strongest apparent toxic effect. MMTD (impurity F) structure was the toxic functional group of cefazolin sodium that caused embryo deformity of zebrafish. 7-ACA structure was the toxic functional group affecting the motor nerve function of zebrafish; the 7-ACA and 3-side chain structure play a role together on the motor nerve system of zebrafish larvae. MMTD and 7-ACA structures were responsible for the toxic cardiac function of cefazolin in zebrafish (Figure 5). This study reveals partly the reason why cefazolin sodium causes a higher proportion of ADR reactions of the cardiovascular and nervous systems than other cephalosporins; and also explains the necessity of quality control on impurities F and G in cefazolin sodium as specific targets in pharmacopeia.

## Ethics statement

This study was carried out in strict accordance with the recommendations in the Regulation for the Management of Laboratory Animals of the Ministry of Science and Technology of China. The protocol was approved by the Committee on the Ethics of Animal Experiments of the Institute of Medicinal Biotechnology, Chinese Academy of Medical Sciences (IMBF20060302).

## Author contributions

CH and JZ conceived and designed the project, analyzed the data. BC, CH, and JZ wrote the manuscript. BC performed the experiments of cefazolin and impurities on the cardiac function of zebrafish and dealt the data; ZG and YL measured the concentrations *in vivo* in zebrafish and dealt data; YZ did the experiments of cefazolin and impurities on larval zebrafish motor behaviors and dealt data; JZ and YH did experiments of embryonic toxicity of zebrafish.

### Conflict of interest statement

The authors declare that the research was conducted in the absence of any commercial or financial relationships that could be construed as a potential conflict of interest.
